# Supplementary material for: Chronic limping in childhood, what else other than juvenile idiopathic arthritis: a case series
Source: Pediatr Rheumatol Online J. 2023 Nov 24;21:142. doi: 10.1186/s12969-023-00927-3 (PMC10668342; doi:10.1186/s12969-023-00927-3)
Supplement: Supplementary file 2 — Supplementary Material 2 [file 12969_2023_927_MOESM2_ESM.doc]

Trieste, November 2nd, 2023

The Editor

*Pediatric Rheumatology*

Dear Editors

Please find submitted the revised version of Case Report entitled "**Chronic limping in childhood, what else other than Juvenile Idiopathic Arthritis: a case series”**.

We have tried to assess all the questions arised from the Reviewers and we appreciate your consideration of this work for publication in *Pediatric Rheumatology.*

All authors meet the journal’s criteria for authorship and have seen and approved this manuscript and have given the necessary attention to ensure the integrity of the work as presented. We do not have any conflict of interest to declare.

As suggested we have provided a “point-to-point” response to reviewers, a track changes version and a cleaned version of the manuscript.

This manuscript is being submitted exclusively to *Pediatric Rheumatology* and is not being considered for publication by any other journal, moreover we declare that there are no prior publications or submissions with any overlapping information.

The undersigning author hopes that in your view, and that of your distinguished reviewers, this manuscript is now of satisfactory quality and is meritorious of publication in the *Pediatric Rheumatology*.

Sincerely,

Andrea Taddio, MD

MAILING ADDRESS

Andrea Taddio, MD

Institute for Maternal and Child Health – IRCCS “Burlo Garofolo”

Via dell'Istria 65/1.

34100. Trieste, Italy

PHONE: +39 040 3785312;

FAX: +39 040 3785458

Email: andrea.taddio@burlo.trieste.it
