# Supplementary material for: Chronic limping in childhood, what else other than juvenile idiopathic arthritis: a case series
Source: Pediatr Rheumatol Online J. 2023 Nov 24;21:142. doi: 10.1186/s12969-023-00927-3 (PMC10668342; doi:10.1186/s12969-023-00927-3)
Supplement: Supplementary file 3 — Supplementary Material 3 [file 12969_2023_927_MOESM3_ESM.docx]

**Chronic limping in childhood, what else other than Juvenile Idiopathic Arthritis: a case series**

**Cristina Tumminelli1, Serena Pastore2, Andrea Taddio1,2***

^1^University of Trieste, Italy,

^2^Institute for Maternal and Child Health Burlo Garofolo (IRCCS), Italy

*Corresponding Author: Andrea Taddio [andrea.taddio@burlo.trieste.it](mailto:andrea.taddio@burlo.trieste.it)

Contributing Authors: [cristina.tumminelli@gmail.com](mailto:cristina.tumminelli@gmail.com); [serena.pastore@burlo.trieste.it](mailto:serena.pastore@burlo.trieste.it)

**Abstract**

**Background:** Limping is a common clinical symptom in childhood; different clinical conditions may lead to limping and the diagnosis of the underlying cause may often be a challenge for the pediatrician.

**Case presentation:** We describe the clinical manifestations, radiological pictures and disease course of other causes of limping in childhood, through a case series of seven cases and a brief discussion of each disease.

**Conclusions:** although trauma is the most common cause of acute limping, when there is no history of traumatic events and the limping has a chronic course, Juvenile Idiopathic Arthritis is usually the most likely clinical diagnosis. However, other some rare conditions should be taken into account if JIA is not confirmed or if it presents with atypical clinical picture.

**Keywords**

Limping child, non-traumatic limping, JIA, differential diagnosis, Chronic Nonbacterial Osteomyelitis, COPA syndrome, CACP syndrome, neuroblastoma, Pigmented villonodular Synovitis, Lyme arthritis

**Background**

Limping in childhood and adolescence is a common clinical problem and it has been estimated that from 1.5 to 3.6 children out of 1000 experiment limping at least once in life (1). In a limping lasts more than 6 weeks, associated with morning stiffness and joint swelling on physical examination, Juvenile Idiopathic Arthritis (JIA) should be suspected (2). The prevalence of JIA in Europe ranges from 3.8 to 400/100,000 and differential diagnosis involves a broad spectrum of diseases. It’s estimated that, over 25% of patients suspected with JIA turns out to be diagnosed with other clinical conditions (3). A prompt recognition may prevent the patient to unnecessary and ineffective therapies. Since a wide range of differential diagnoses of JIA is reported in the literature, we believe that it’s important to discuss some less frequent causes of limping that could mimic JIA.

**Case presentation**

**Case 1**

A 2-year-old girl was admitted for a 6-months history of right intermittent limping and arthralgia. She was a preterm, born at 27 weeks, with mild bronchopulmonary dysplasia and bilateral renal dysplasia. Her family history was positive for a mother with adult-onset Still's disease and a father with end- stage renal failure. The physical examination was unremarkable, except for limitation of motion of right hip and both wrists. Blood tests showed a normal cell blood count (CBC), with an increase in erythrocyte sedimentation rate (ESR) and hypergammaglobulinemia (IgG: 1430 mg/dl; IgA: 450 mg/dl; IgM: 214 mg/dL). Her renal function was normal (creatinine 0.46 mg/dl, urea 30 mg/dl), and urinalysis was negative for proteinuria and hematuria. Autoimmunity markers showed positivity to Anti-Nuclear Antibody (ANA) 1:160. Wrists and hips X- rays were normal; the osteo-articular ultrasound (US) showed an effusion of both radiocarpal joints with synovial thickening (fig.1). The abdominal US performed was unremarkable. To better understand her past medical condition, a chest X-Ray first, and a pulmonary computerized tomography (CT) were performed, which revealed ground glass opacities with diffuse interstitial thickening (fig.2). Secondary analysis showed positivity to Anti- cyclic citrullinated peptide (CCP) > 340 U/mL and Rheumatoid Factor (RF): 574 UI/mL, while Extractable Nuclear Antigen Antibodies (ENA), Anti-native DNA Antibodies (nDNA) and Antineutrophil Cytoplasmic Antibodies (ANCA) were negative. Mantoux test and QuantiFERON test were negative, ruling out tuberculosis. The Neuron-specific enolase (NSE) resulted in a positive value (56.7mcg/L); however, urinary homovanillic and vanillylmandelic acids were negative. The clinical picture, characterized by early-onset polyarthritis, ANA and FR positivity and pulmonary involvement, was strongly suggestive of Coatomer Protein Subunit Alpha (COPA) syndrome, that was confirmed by genetic analysis (mutation in heterozygous *COPA gene*, variant c.841C>T p. Arg281Trp) and a treatment with Jak inhibitor was started.

**COPA Syndrome**

COPA syndrome is a rare autosomal dominant disorder (prevalence of <1 / 1000 000), with variable penetrance, caused by mutation in the gene encoding COPα protein, which is part of the coatomer protein complex I (COPI). In healthy person, coatomer helps to mediate retrograde movement of vesicles from the Golgi apparatus to the endoplasmic reticulum (ER), but when mutated it induces an increasing in ER protein synthesis, a pronounced ER stress response and the activation of inflammatory cascade. Recent hypotheses have emerged regarding the fact that COPA syndrome may also involve the STING pathway, which would lead to an enhancement of type I-IFN signaling. This would be in line with the clinical similarity between COPA syndrome and STING-associated vasculopathy with onset in infancy (4). The majority of patients have an early onset disease, usually before 5 years of age with pulmonary disease always present, although clinical severity may vary a lot among patients; lung involvement develops during time, with worsening pneumopathy and tendency to develop alveolar hemorrhages or cystic formations. Renal involvement can also be severe and it’s due to immune-mediated glomerulonephritis or also tubulopathy (5). Musculoskeletal manifestations are a common feature; approximately 86% of the patients have polyarticular arthritis affecting the knees and the interphalangeal joints of the hands. A descriptive cohort study of 14 patients with COPA syndrome showed that 35% out of 14 subjects were initially diagnosed with JIA (6). Inflammatory markers are elevated, including CRP, ESR and immunoglobulins. Additional elements, suggesting an immune dysregulation, include positive ANA as well as RF and anti-CCP. It has also been described as a diffuse interstitial-pulmonary neuroendocrine cell hyperplasia which is responsible for NSE elevation (7). However, no single autoantibody may be considered as marker of disease. Genetic analysis allows to identify the mutation responsible for the disease. Regarding therapy, management is based on systemic corticosteroids during the exacerbations of diseases and immunosuppressive drugs for the maintenance therapies. Recently, baricitinib, an inhibitor of JAK-1 and JAK-2, has been proven to improve the disease course, since the patients have an increased type 1 interferon pathway (6).

**Case 2**

A previously healthy 2-year-old boy was evaluated for a 2- month history of persistent right limping. His medical and his family history were unremarkable. The physical examination was unremarkable, except for a mild swelling of his right ankle and a painful range of motion (ROM) limitation. The laboratory exams including CBC, ESR, CPR, renal and hepatic function were normal. The ankle X-Ray was normal, while US showed a mild intraarticular fluid collection. Oligoarticular JIA was suspected and naproxen was started. One month later, since no improvement was reported, the child underwent a new ankle X-Ray which revealed a small lytic bone lesion. Slight anemia (Hb 11 g/dl) and a high ESR (60 mm/h) were found on laboratory exams. The NSE and 24 h urine vanillylmandelic acid were increased (respectively 70.01 ng/mL and 50 μmol/24 h). A diagnostic bone marrow aspiration demonstrated histological features consistent with neuroblastoma and lytic bone lesion was consistent with metastases. The CT scan found the primary lesion in the left adrenal gland and the patient was referred to the oncologist department to start the proper treatment.

**Neuroblastoma**

Neuroblastoma is the most common extracranial solid tumor in childhood originating from neural crest cell with a prevalence of 1-5/10000. Several genomic alterations are responsible of the neuroblastoma, for example amplification of MYC-N, mutations of ALK or chromosomic deletions. About 65% occur in the abdomen, often from the adrenal gland (46% of cases) (8). Its classical presentation is an abdominal painful palpable mass and other symptoms depending on primary tumor size and metastatic sites. The most common areas of metastasis are bones, bone marrow and liver and about 25% of clinical presentations are secondary to bone localization. However, limping is a rare symptom and often is misdiagnosed as arthritis (9). Chu et al. reported two cases of neuroblastoma with bone involvement, previously diagnosed as JIA or septic arthritis (10). The patients with neuroblastoma have usually lot of pain but very mild clinical signs (11). Diagnosis is based on imaging, although tissue histology is generally necessary to confirm. Skeletal lesions may be osteolytic or may present as pathological fracture. However, X-Ray abnormalities are rarely present at onset, and also laboratory exams could be normal. The overall outcomes depend on prompt diagnosis and the treatment consists of chemotherapy, sometimes adrenalectomy and post-consolidation therapy.

**Case 3**

A 10-year-old girl presented with a 3-month long lasting history of left limping and painful ankle without any other significant clinical complain. Her past medical history and her family history were unremarkable. The physical examination was unremarkable except for a swollen left ankle with a limitation of ROM. Laboratory exams showed an increase in ESR (48 mm/h) with normal CRP, CBC, liver and renal function and a positive for ANA 1:160. Oligoarticular JIA was suspected and clinical remission after intra-articular corticosteroid injection was achieved. Two years later, the patient developed significant painful swollen on the right side of the jaw. The ESR was elevated (60mm/h). On US edema of masseter muscle was found with no sign of temporomandibular joint effusion. She was treated for myositis with nonsteroidal anti- inflammatory drugs (NSAIDS), with benefit until its suspension. A magnetic resonance (MRI) of the jaw was performed, showing edema of the right masseter muscle, small areas of hypointensity and hypertrophy of right mandibular ramus and enhancement of the endosteal signal. Mandibular bone involvement was also confirmed by CT (fig 3a) while an X-Ray showed a bone osteolytic lesion on the ankle (fig. 3b). An ankle bone biopsy was performed, which showed a sterile inflammatory infiltrate with neutrophils and plasmacells, without neoplastic cells. Immunochemistry was negative for the presence of S100 and CD1, excluding therefore a histiocytosis. Clinical history, physical examination and histopathological pattern were highly suggestive of Chronic Nonbacterial Osteomyelitis (CNO). A full-body MRI was performed but no other lesions were found. A therapy with NSAIDs was started in association with TNF-α blockers with clinical improvement.

**Chronic Nonbacterial Osteomyelitis**

CNO is a rare auto-inflammatory disorder with a prevalence of 1-9/1000000. It is characterized by single or multiple bone lesions which cause CNO insidious recurrent bone pain in the morning and during the night. Moreover, an association with other inflammatory conditions such as inflammatory bowel disease or psoriasis is reported in the literature (12). Pain can be associated with warmth and/or localized swelling and loss of function, because in 30% of cases CNO involves the adjacent joint. Any site of the skeleton could be involved, but long bones are the most frequent sites. Jaw osteomyelitis is very suggestive of CNO, as well as the localization in the sternum, clavicle, while skull is more frequent in histiocytosis (13). The median age at diagnosis in pediatric patients is 10 years, with a female-to-male ratio of 2:1. Diagnostic delay is common in pediatric CNO, with a mean interval of 12 months between symptom onset and diagnosis in most pediatric studies, and this is related to the absence of pathognomonic laboratory markers. JIA, infective osteomyelitis, malignancy and vitamin C deficiency could be differential diagnoses (14). CNO is a diagnosis of exclusion based on imaging (such as x- rays, bone scan or MRI), bone biopsy, and laboratory blood tests, although these exams may be normal in most patients, or can show non- specific elevated markers of inflammation (ESR, CRP). NSAIDs are the first-choice treatment. Corticosteroids, methotrexate, bisphosphonates, TNFα-inhibitors and IL-1 blockers have also been used with some benefits and the choice of the second line treatment depends on bone lesions localizations, presence of systemic features and patients' clinical conditions (13).

**Case 4**

A 12-year-old boy presented a 3-months history of right limping and painful right knee. His past medical history and familiar history were unremarkable. The physical examination was unremarkable except for a swallen right knee. Laboratory tests, including CBC, CRP, ESR, renal, hepatic function and autoimmunity markers, were normal. The US showed an effusion in the suprapatellar capsule of the left knee. An oligoarticular JIA was suspected and after intra-articular corticosteroid injection oral therapy with NSAIDs was started. Two months later, the right knee effusion worsened with limited ROM. The knee X-Ray performed, revealed multiple radiopaque, round, loose bodies within the joint (fig.4a) consistent with a diagnosis of synovial chondromatosis, confirmed by histological findings after arthroscopic synovectomy that showed mature hyaline cartilage with fibrosis and microscopically chondrocytes (fig. 4b).

**Synovial Chondromatosis**

Synovial chondromatosis is a benign condition characterized by metaplasia of synovial cells forming cartilaginous bodies and intra-articular loose subsynovial tissue bodies especially in large joints (knees, hips and shoulders). It’s rare in childhood (prevalence of 1/100000) in which may mimic oligoarticular JIA (15). It’s considered as a benign tumor and retrospective studies from adults describe a low risk of developing in chondrosarcoma. Plain radiography and MRI imaging are considered the gold standard for diagnosis; however, they can be initially negative because of the same signal intensity between fluid and non-mineralized synovium (16). The response to NSAIDs is reported only in the early phases, while surgical arthroscopy remains the best treatment available with good clinical outcome.

**Case 5**

A previously healthy 3-year-old child was evaluated for a 3-month history of asthenia, left limping and bilateral knees effusion, more evident on the left. A progressive increase in child’s abdominal circumference was also reported. Her family history was remarkable for Hodgkin lymphoma in her mother and cardiomyopathy in her first cousin. On physical examination, a mild tachycardia (heart rate 135-140 bpm) was detected. Heart and lung examination were unremarkable; the abdomen was distended, not painful, with palpable liver and broad tympany on percussion. Swelling of the knees without other inflammatory signs and bilateral congenital trigger fingers of the hands were reported. Blood tests showed neutrophilic leukocytosis and thrombocytosis (WBC 12.520/mmc, PLT 500.000/mmc) with mild increase in gamma- glutamyl transferase. All other laboratory exams, including coagulation, electrolytes, CRP, ESR, and renal function were normal. The abdominal US showed an intrabdominal effusion and the chest X-Ray reveled an increase in cardiothoracic ratio (fig.5). For this reason, an echocardiography was performed and restrictive cardiomyopathy due to pericardial effusion was found. The complexity of the clinical picture led to perform genetic analysis, that reveled a mutation in the *PRG4 gene* and diagnosis of Camptodactyly- arthropathy-coxa vara-pericarditis (CACP) syndrome was made. A mild bilateral coxa vara was highlighted by a pelvis and lower limbs X-Ray, performed after the outcome of the genetic investigations. The patient was therefore referred to the cardiology department and ibuprofen was prescribed in associations with intensive physiotherapy.

**CACP Syndrome**

CACP syndrome is a rare recessive disorder (prevalence of <1/1000000) caused by a mutation in the *PRG4 gene* coding for a lubricating proteoglycan of the surface of articular cartilage. The loss of function mutation causes progressive joints deformity due to a hyperplasic process of the synoviocytes, responsible for the typical features (camptodactyly, arthropathy and coxa vara). In about 20% of cases, in addition to joint involvement, pericardial involvement is described, suggesting that CACP syndrome may also be due to a regulatory dysfunction in the proliferation of serosal cells (17). This condition is likely underdiagnosed and sometimes misdiagnosed as polyarticular or systemic JIA when pericardial involvement is present. However, in contrast to JIA, there is no joint inflammation, and therefore, there is little response to anti-inflammatory therapy; In addition, MRI allows to find the typical acetabular cysts (18). The therapeutic management is only symptomatic in association with physiotherapy and multidisciplinary approach (19).

**Case 6**

A previously healthy 16-year-old boy was evaluated for 6-month history of left limping with recurrent left knee arthritis treated with intra-articular corticosteroid injection with transient benefit but, afterwards, swelling recurrence. His past medical history and familiar history were unremarkable. He was evaluated at our Institute during the third episode of swollen knee, 2 months after the first episode. Despite the swelling, he did not report morning stiffness or joint pain. The physical examination was unremarkable except for a warm, swallen right knee with limited ROM. CBC, ESR, CRP and autoantibody profiles (ANA and anti-DNA) were normal. Knee US showed significant intra-articular effusion and synovial thickening, with no positivity to color-doppler signal. Oligoarticular JIA was suspected and a knee arthrocentesis was performed with 270 ml of bloody fluid drained (fig. 6). Synovial fluid analysis showed a mild increase in WBC count (20.267cells/uL), with negative bacterial culture. One month after arthrocentesis, the joint picture appeared to have improved; however, slight intra- articular effusion persisted on US. Based on the pattern of recurrent arthritis, although the boy denied any travel to endemic areas or tick bites, second level analysis were performed that showed seropositivity for Borrelia Burgdorferi, confirmed by positive immuno-blot; diagnosis of Lyme arthritis (LA) was made and antibiotic therapy with Amoxicillin 50 mg / kg/ day for 28 days was started.

**Lyme arthritis**

LA is a manifestation of infection with Borrelia burgdorferi spirochete. It’s widespread in North America, but it’s infrequent in Europe, although an increase in its incidence has also been recorded, ranging from 0.14 to 1.4 cases/1000 habitants/year, due to the greater diffusion of the vector (20). Lyme disease usually begins with an expanding skin lesion, known as erythema migrant (stage 1) that, if untreated, can be followed within days to weeks, by early disseminated infection, responsible for neurological and cardiac involvement (stage 2). Late infection occurs after months and it’s characterized by arthritis or acrodermatitis chronica atrophicans (stage 3). However, LA may be not associated with a history of tick bite and / or erythema migrant, so arthritis can be the first manifestation. At the onset, LA usually affects large joints with recurrent swelling often associated with limited ROM but little or no pain and the knee is the most affected joint. The ankle is the second one and the picture may mimic an oligoarticular JIA (21). Serological testing is the only available method to support a diagnosis of Lyme borreliosis and it’s based on a 2- step test using serological screening and confirmation with immunoblot or Polymerase Chain Reaction. Although a course of oral antibiotics leads to resolution in over 70% of cases, in a smaller proportion of patients, an immunological mechanism leads to chronic arthritis, indistinguishable from JIA (22).

**Case 7**

A 14-year-old boy was referred to our Service for a 3-year history of swollen right knee unresponsive to intra-articular corticosteroid injections. His past medical history and his family history were unremarkable. The physical examination was unremarkable except for swelling of the right knee. The laboratory tests revealed always normal CBC and negative inflammatory markers and secondary analysis showed RF, ANA, HLA- B27 and Lyme disease, negative results. A knee MRI showed a large effusion and hyperplasia of the synovium, with no bone erosions or lytic lesions (fig. 7). The arthrocentesis performed resulted in aspiration of a small quantity of bloody fluid. The presence of a monoarticular arthritis, unresponsive to therapy, with no bone erosions despite the long history, led us to perform an arthroscopy with synovectomy. On histological examination, proliferation of mononuclear cells, multinucleated giant cells and macrophages overloaded with hemosiderin were noted and diagnosis of Pigmented Villonodular Synovitis (PVS) was made.

**Pigmented Villonodular Synovitis**

PVS is a synovial proliferative lesion rare in children but more frequent in young adults with a prevalence of 1.8/1000000, in which there is often a monoarticular knee involvement with a marked swelling without pain or inflammation. Pathogenesis is unknown, but it results in a synovial hyperplasia and accumulation in the synovia of histiocytes, multinucleated giant cells, and macrophages overloaded with hemosiderin. This accumulation gives a typical picture on MRI, which represents the gold standard for the diagnosis showing focal irregular high signal intensity lesions on T2-weighed images (23). However, the slow growth and low intensity of pain at early-stage may lead to a delayed diagnosis or a misdiagnosis with JIA or other rheumatologic disorders (24). Diagnostic confirmation is by histology after total synovectomy, which represents the main treatment (25).

**Case 8**

A 2-year-old girl presented with a right limping. Her past medical history and her family history were unremarkable. The physical examination was unremarkable except for a swollen, painful right knee with a limitation of ROM. Laboratory exams showed an increase in ESR (40 mm/h) with normal CRP, CBC, liver and renal function and a positive for ANA 1:1280. Oligoarticular JIA was suspected and clinical remission after intra-articular corticosteroid injection was achieved. However, the child presented a relapse of arthritis in the following months, in her left ankle and left knee for which medicated arthrocentesis was performed and therapy with methotrexate was started with subsequent clinical remission.

**JIA**

JIA is the most common rheumatologic disease in children, with a prevalence ranging from 3.8 to 400/100,000 (3). Limping could be a symptom of JIA in children, especially in females. The most frequently affected joints are the knees and ankles with swelling, local warmth, and a limited ROM on physical examination (26). Diagnosis is clinical, and laboratory tests are usually normal. ANA may be normal and they are used for uveitis risk stratification. NSAIDs and corticosteroid joint injections are the main treatment for JIA. Second level drugs such as DMARDs or biologic agents for example anti-TNF drugs, are indicated in children that have severe or progressive arthritis (28).

**Discussion and Conclusions**

In this article, we described some important causes of chronic limping, that, although rare, clinicians must suspect in presence of specific symptoms and signs (table 1) and a more common case, the JIA. However, a persistent limping is not necessarily a sign of JIA, and the differential diagnosis involves a broad spectrum of rheumatic and non-rheumatic diseases (table 2). An early onset arthritis with systemic involvement and a positive family history could be secondary to genetic conditions (*case 1* and *case 5*), while a history of recurrent monoarticular arthritis should arouse suspicion of LA (*case 6),* even in the absence of tick bite history. Except in the systemic onset, laboratory tests including blood cell counts and inflammatory markers are usually normal in JIA. An alteration in laboratory tests, such as anemia can be found in presence of malignancy. It’s imperative to remember that tumors may have bone involvement, as in the case of neuroblastoma metastases (*case 2*) and that the lesions may initially not be evident and blood tests may be normal. Additional testing, such as Lyme serology or tumor markers may be considered, based on the potential differential diagnosis. Furthermore, the diagnostic approach on a limping child often include plain inferior limb X-Rays and joint US for the detection of focal anomalies or articular effusion respectively. Sometimes, repeating an X-Ray is mandatory in frequent relapse *(case 3).* However, if there is discrepancy between important symptoms and mild clinical signs of arthritis considers malignancy even if X-Ray is negative and, in that case, consider MRI *(case 2)*. MRI plays a major role for diagnosis of potential underlying etiologies (*cases 3 and 4*). Finally, the arthrocentesis is a procedure that may be necessary for some conditions thanks to its diagnostic and therapeutic value. Macroscopically, synovial fluid analysis can provide information in terms of quantity, the possible presence of hemarthrosis and its viscosity (*cases 6 and 7).* Moreover, an analysis of cellularity is useful to investigate an inflammatory condition and also a histological analysis of synovial tissue may help to orientate in specific diagnosis *(case 7)*. In conclusion, we suggest thinking about alternative diagnoses, although rare, when you are in presence of persistent limping and atypical JIA

**List of abbreviations**

Juvenile Idiopathic Arthritis (JIA); Erythrocyte Sedimentation Rate (ESR); Neuron-Specific Enolase(NSE); Anti-Nuclear Antibody (ANA); Ultrasound (US); Anti-Cyclic Citrullinated Peptide (CCP); Rheumatoid Factor(RF); Extractable Nuclear Antigen Antibodies (ENA); Anti-Native DNA Antibodies (nDNA); Antineutrophil Cytoplasmic Antibodies (ANCA); Computerized Tomography (CT); Coatomer Protein Subunit Alpha (COPA); Endoplasmic Reticulum (ER); C-Reactive Protein (CRP); Range Of Motion (ROM); Nonsteroidal Anti-Inflammatory Drugs (NSAIDS); Magnetic Resonance Imaging (MRI); Chronic Nonbacterial Osteomyelitis (CNO); White Blood Cells (WBC); Platelets (PLT); Camptodactyly-Arthropathy-Coxa Vara-Pericarditis (CACP);Lyme Arthritis (LA); Pigmented Villonodular Synovitis (PVS).

**Declarations**

**Ethics approval and consent to participate:** Not applicable

**Consent for publication:** informed consent was obtained from all parents of children involved

**Availability of data and materials:** Not applicable

**Competing interests:** the authors declare that they have no competing interests

**Funding:** This work was supported by the Italian Ministry of Health, through the contribution given to the Institute for Maternal and Child Health IRCCS Burlo Garofolo- Trieste, Italy - RC 34\18

**Authors' contributions:** all authors contributed to the conception and design of the study. CT wrote the first draft of the manuscript. All authors wrote sections of the manuscript. All authors contributed to the review of the manuscript, they read and approved the submitted version

**Acknowledgements:** Not applicable

**References**

1. Lázaro Carreño MI, Fraile Currius R, García Clemente A. Non-traumatic limping in Paediatric Emergencies: Epidemiology, evaluation and results. Rev Esp Cir Ortop Traumatol (Engl Ed). 2018 Mar-Apr;62(2):127-133. English, Spanish. doi: 10.1016/j.recot.2017.10.004. Epub 2017 Nov 12. PMID: 29138043.
2. Jeamsripong S, Charuvanij S. Features distinguishing juvenile idiopathic arthritis among children with musculoskeletal complaints. World J Pediatr. 2020 Feb;16(1):74-81. doi: 10.1007/s12519-018-0212-0. Epub 2018 Nov 29. PMID: 30498888.
3. Freychet C, Lambert C, Pereira B, Stephan JL, Echaubard S, Merlin E, Chausset A. Medical pathways of children with juvenile idiopathic arthritis before referral to pediatric rheumatology centers. Joint Bone Spine. 2019 Nov;86(6):739-745. doi: 10.1016/j.jbspin.2019.04.014. Epub 2019 May 20. PMID: 31121314.
4. Wobma H, Shin DS, Chou J, Dedeoğlu F. Dysregulation of the cGAS-STING Pathway in Monogenic Autoinflammation and Lupus. Front Immunol. 2022 May 27;13:905109. doi: 10.3389/fimmu.2022.905109. PMID: 35693769; PMCID: PMC9186411.
5. Taveira-DaSilva AM, Markello TC, Kleiner DE, Jones AM, Groden C, Macnamara E, Yokoyama T, Gahl WA, Gochuico BR, Moss J. Expanding the phenotype of COPA syndrome: a kindred with typical and atypical features. J Med Genet. 2019 Nov;56(11):778-782. doi: 10.1136/jmedgenet-2018-105560. Epub 2018 Nov 1. PMID: 30385646; PMCID: PMC6860403.
6. Tsui JL, Estrada OA, Deng Z, Wang KM, Law CS, Elicker BM, Jones KD, Dell SD, Gudmundsson G, Hansdottir S, Helfgott SM, Volpi S, Gattorno M, Waterfield MR, Chan AY, Chung SA, Ley B, Shum AK. Analysis of pulmonary features and treatment approaches in the COPA syndrome. ERJ Open Res. 2018 Jun 27;4(2):00017-2018. doi: 10.1183/23120541.00017-2018. PMID: 29977900; PMCID: PMC6019741.
7. Vece TJ, Watkin LB, Nicholas S, Canter D, Braun MC, Guillerman RP, Eldin KW, Bertolet G, McKinley S, de Guzman M, Forbes L, Chinn I, Orange JS. Copa Syndrome: a Novel Autosomal Dominant Immune Dysregulatory Disease. J Clin Immunol. 2016 May;36(4):377-387. doi: 10.1007/s10875-016-0271-8. Epub 2016 Apr 5. PMID: 27048656; PMCID: PMC4842120.
8. Newman EA, Abdessalam S, Aldrink JH, Austin M, Heaton TE, Bruny J, Ehrlich P, Dasgupta R, Baertschiger RM, Lautz TB, Rhee DS, Langham MR Jr, Malek MM, Meyers RL, Nathan JD, Weil BR, Polites S, Madonna MB; APSA Cancer committee. Update on neuroblastoma. J Pediatr Surg. 2019 Mar;54(3):383-389. doi: 10.1016/j.jpedsurg.2018.09.004. Epub 2018 Sep 19. PMID: 30305231.
9. Parmar R, Wadia F, Yassa R, Zenios M. Neuroblastoma: a rare cause of a limping child. How to avoid a delayed diagnosis? J Pediatr Orthop. 2013 Jun;33(4):e45-51. doi: 10.1097/BPO.0b013e318279c636. PMID: 23653041.
10. Chu CM, Rasalkar DD, Hu YJ, Cheng FW, Li CK, Chu WC. Clinical presentations and imaging findings of neuroblastoma beyond abdominal mass and a review of imaging algorithm. Br J Radiol. 2011 Jan;84(997):81-91. doi: 10.1259/bjr/31861984. PMID: 21172969; PMCID: PMC3473807.
11. Trapani S, Grisolia F, Simonini G, Calabri GB, Falcini F. Incidence of occult cancer in children presenting with musculoskeletal symptoms: a 10-year survey in a pediatric rheumatology unit. Semin Arthritis Rheum. 2000 Jun;29(6):348-59. doi: 10.1053/sarh.2000.5752. PMID: 10924020.
12. Hofmann SR, Kapplusch F, Girschick HJ, Morbach H, Pablik J, Ferguson PJ, Hedrich CM. Chronic Recurrent Multifocal Osteomyelitis (CRMO): Presentation, Pathogenesis, and Treatment. Curr Osteoporos Rep. 2017 Dec;15(6):542-554. doi: 10.1007/s11914-017-0405-9. PMID: 29080202; PMCID: PMC5705736.
13. Kim SM, Lee SK. Chronic non-bacterial osteomyelitis in the jaw. J Korean Assoc Oral Maxillofac Surg. 2019 Apr;45(2):68-75. doi: 10.5125/jkaoms.2019.45.2.68.
14. Robertson LP, Hickling P. Chronic recurrent multifocal osteomyelitis is a differential diagnosis of juvenile idiopathic arthritis. Ann Rheum Dis. 2001 Sep;60(9):828-31. PMID: 11502607; PMCID: PMC1753836.
15. Yothakol N, Charuvanij S, Siriwanarangsun P, Lertwanich P, Muangsomboon S, Sukharomana M. Synovial osteochondromatosis mimicking juvenile idiopathic arthritis in an adolescent: a case-based review. Clin Rheumatol. 2022 Aug;41(8):2571-2580. doi: 10.1007/s10067-022-06224-w. Epub 2022 May 31. PMID: 35641775; PMCID: PMC9154203.
16. Yi RB, Gong HL, Arthur DT, Wen J, Xiao S, Tang ZW, et al. Synovial chondromatosis of the hip joint in a 6 year-old child: A case report. World J Clin Cases. 2022 Jan 14;10(2):685- 690. doi: 10.12998/wjcc.v10.i2.685
17. Kakkar RM, Soneji S, Badhe RR, Desai SB. Camptodactyly-arthropathy-coxa vara-pericarditis syndrome: important differential for juvenile idiopathic arthritis. J Clin Imaging Sci. 2013 Jun 29;3:24. doi: 10.4103/2156-7514.114211. PMID: 24083061; PMCID: PMC3779395.
18. Hugosson C, Bahabri S, McDonald P, al-Dalaan A, al-Mazyed A. Radiological features in congenital camptodactyly, familial arthropathy and coxa vara syndrome. Pediatr Radiol. 1994;24(7):523-6. doi: 10.1007/BF02015019.
19. Madhusudan S, Gupta A, Prakash M, Matta D, Suri D, Singh S. Camptodactyly-arthropathy- coxa vara-pericarditis (CACP) syndrome: a mimicker of juvenile idiopathic arthritis. Scand J Rheumatol. 2016 Jan;45(1):77-78. doi: 10.3109/03009742.2015.1085085.
20. Nazzi F, Martinelli E, Del Fabbro S, Bernardinelli I, Milani N, Iob A, Pischiutti P, Campello C, D'Agaro P. Ticks and Lyme borreliosis in an alpine area in northeast Italy. Med Vet Entomol. 2010 Sep;24(3):220-6. doi: 10.1111/j.1365-2915.2010.00877.x. Epub 2010 May 30. PMID: 20534008.
21. Orczyk K, Świdrowska-Jaros J, Smolewska E. When a patient suspected with juvenile idiopathic arthritis turns out to be diagnosed with an infectious disease - a review of Lyme arthritis in children. Pediatr Rheumatol Online J. 2017 May 8;15(1):35. doi: 10.1186/s12969-017-0166-0. PMID: 28482848; PMCID: PMC5422956.
22. Huppertz HI, Karch H, Suschke HJ, Döring E, Ganser G, Thon A, Bentas W. Lyme arthritis in European children and adolescents. The Pediatric Rheumatology Collaborative Group. Arthritis Rheum. 1995 Mar;38(3):361-8. doi: 10.1002/art.1780380310. PMID: 7880190.
23. Stephan SR, Shallop B, Lackman R, Kim TWB, Mulcahey MK. Pigmented Villonodular Synovitis: A Comprehensive Review and Proposed Treatment Algorithm. JBJS Rev. 2016 Jul 19;4(7):e3. doi: 10.2106/JBJS.RVW.15.00086.
24. Turkucar S, Makay B, Tatari H, Unsal E. Pigmented villonodular synovitis: Four pediatric cases and brief review of literature. J Postgrad Med. 2019 Oct-Dec;65(4):233-236. doi: 10.4103/jpgm.JPGM_305_19. PMID: 31611431; PMCID: PMC6813690.
25. Sun H, Ju XD, Huang HJ, Zhang X, Wang JQ. Clinical outcomes of endoscopic synovectomy with adjuvant radiotherapy of pigmented villonodular synovitis of the hip: a case series of single center. BMC Musculoskelet Disord. 2022 Mar 2;23(1):192. doi: 10.1186/s12891-022-05141-y. PMID: 35236301; PMCID: PMC8889717.
26. Santili C, Júnior WL, Goiano Ede O, Lins RA, Waisberg G, Braga Sdos R, Akkari M. LIMPING IN CHILDREN. Rev Bras Ortop. 2015 Dec 8;44(4):290-8. doi: 10.1016/S2255-4971(15)30156-7. PMID: 27022509; PMCID: PMC4799062.
27. Zamora-Legoff JA, Krause ML, Crowson CS, Muskardin TW, Mason T, Matteson EL. Treatment of patients with juvenile idiopathic arthritis (JIA) in a population-based cohort. Clin Rheumatol. 2016 Jun;35(6):1493-9. doi: 10.1007/s10067-016-3190-4. Epub 2016 Jan 29. PMID: 26825065; PMCID: PMC4870092.

**Figure 1** Longitudinal scan showing effusion in the capsule of the left coxo-femoral joint (blue arrow) with synovial thickening (orange arrow)

**Figure 2** Chest X-ray with diffuse interstitial thickening (a); pulmonary CT with diffuse marked

thickening of the pulmonary interstitium with ground-glass areas especially at the lower lobes. At the

subpleural level, bilaterally, tiny cystic formations associated with bronchiolectasias (b)

**Figure 3** CT cone beam: inhomogeneous bone thickening of the branch and of the right mandibular

angle associated with erosive areoles (a); left ankle X-Ray with osteolytic lesion of the tibial metaphysis (b)

**Figure 4** X-Ray of the right knee: multiple radiopaque, round, loose bodies within the joint (a)

Arthroscopy during synovectomy: multiple white, shiny loose bodies were within the articular space (b)

**Figure 5** Chest x-ray: flask appearance of the cardiac shadow due to pericardial effusion

**Figure 6** 270 cc of blood serum from knee arthrocentesis performed after 2 months of limping

**Figure 7** MRI of the right knee with proton density-weighted axial view showing hyperplasia of the

synovium and low signal intensity indicating hemosiderin (arrow)

**Table 1. The main clinical and laboratorial characteristics of each disease that were described compared to those of JIA**

|  | **JIA** | **COPA**  **syndrome** | **Neuroblastoma** | **CNO** | **Synovial**  **chondromatosis** | **CACP**  **syndrome** | **LA** | **PVS** |
| --- | --- | --- | --- | --- | --- | --- | --- | --- |
| **Age Of Onset** | Oligoarticular 1-3 Yrs  FR- Polyarticular Biphasic Trend Between 2-4  Years And 6 -12 Years  FR+ Polyarticular 13-16 Years  Systemic 2-6 Years  ERA > 6 Years  Psoriatic Biphasic Trend Between 6-  8 Years And 12-15 Years | Before 5 yrs of age | Before 6 yrs of age | Before 10 yrs of age | Between 13-15 yrs of age | 2 yrs of age | 12-17 yrs of age | Average 9 yrs of age |
|  |  |  |  |  |  |  |  |  |
|  |  |  |  |  |  |  |  |  |
|  |  |  |  |  |  |  |  |  |
|  |  |  |  |  |  |  |  |  |
|  |  |  |  |  |  |  |  |  |
| **Prevalence** | 3.8 to 400/100,000 | <1 /  1000 000 | 1-5 / 10 000 | 1-9 / 1 000 000 | 1/100 000 | <1 /  1000 000 | 2.3/100 | 1.8/1000000 |
| **Main Localization** | Oligoarticular Large Joints  Polyarticular Small Joints  Systemic and Psoriatic  Large And Small Joints  ERA Large Joints of the Lower Extremities | Polyarticular Arthritis  Particular Knees  And interphalang -eal Joints | Primary Tumor in Adrenal Gland  Metastatic Sites: Bones, Bone Marrow, And Liver | Long Bones | Large Joints: Knees, Hips Shoulders | Camptoda ctyly  Arthropat hy Of Large Joints Coxa Vara  Deformity | Large Joints Arthritis | Knee involvement |
| **Pain** | ++/- | +++ | Dispropor-tionate Pain Level In Contrast To A Mild Swelling | ++  Recurrent pain | + | + | Low Pain Level in Contrast to Major  Swelling | - |
| **Inflammatory Markers** | ++/- | +++ | + | + + | - | - | + | + |
| **Extra- articular Manifestation** | Enthesitis (ERA)  Dactylitis (ERA and Psoriatic Type)  Uveitis (all subtypes)  Hepatosplenomeg -aly, Lymphadenopath, Rash, Synovitis (Systemic) | Renal Pulmonary Involvement | Abdominal Painful Palpable Mass Anemia  Loss of Weight | Possible combinate with Inflammatory Conditions | None | Pericardia l Involvem ent | Erythema Migrant Nervous system and Cardovas-cular system | None |
|  |  |  |  |  |  |  |  |  |
|  |  |  |  |  |  |  |  |  |
| **Confirmatory Diagnosis** | No Specific Tests | Genetic Analysis | Histological examination | No Specific Tests | MR Imaging +  Histological Findings | Genetic Analysis | Clinical findings+  Serological Testing | Histological Findings |
|  |  |  |  |  |  |  |  |  |

YRS- years, RF – rheumatoid factor, ERA - enthesitis-related arthritis, CNO -Chronic Nonbacterial Osteomyelitis, LA- lyme arthritis, PVS- Pigmented Villonodular Synovitis

**Table 2. Causes of limping in children by etiology**

|  | **More common causes of limping** | **Less common causes of limping** |
| --- | --- | --- |
| **Infectious** | Septic Arthritis | Lyme arthritis |
|  | Sacroiliitis | Tuberculosis |
|  | Osteomyelitis | Discitis |
|  |  | Psoitis |
| **Inflammatory** | Transient Synovitis | COPA syndrome |
|  | ﻿﻿JIA | Chronic nonbacterial osteomyelitis |
|  | ﻿﻿Reactive arthritis | Rheumatic fever |
| **Neoplastics** | Leukemia | Osteosarcoma |
|  | ﻿﻿Neuroblastoma | Ewing's sarcoma |
|  | ﻿﻿ Osteoid osteoma | Synovial chondromatosis |
|  |  | Pigmented villonodular synovitis |
| **Degenerative-Mechanics** | Fractures | CACP syndrome |
|  | Developmental Dysplasia of the Hip | Vitamin C Deficiency |
|  | Slipped capital femoral epiphysis | Hypervitaminosis A |
|  | Osteochondrosis | Psuedorheumatoid-arthropathy of childhood |
|  | Legg-Calvé-Perthes Disease | Mucopolysaccharidosis type I |
