# Supplementary material for: Chronic limping in childhood, what else other than juvenile idiopathic arthritis: a case series
Source: Pediatr Rheumatol Online J. 2023 Nov 24;21:142. doi: 10.1186/s12969-023-00927-3 (PMC10668342; doi:10.1186/s12969-023-00927-3)
Supplement: Supplementary file 1 — Supplementary Material 1 [file 12969_2023_927_MOESM1_ESM.docx]

Dear Editor,

On behalf of the authors, I wish to thank you and the reviewers for your consideration and comments on our manuscript entitled “Chronic limping in childhood, what else other than JIA: a case series”.

Reviewers’ comments have been carefully considered and the manuscript has been revised accordingly.

A point-by-point answer to the reviewers’ comments is provided below:

**Reviewer 1:**

**The authors present 7 interesting cases, all related to the diagnostic workup in pediatric rheumatology.
The manuscript is written well, adressing the presented topics in a comprehensive style.
Nevertheless the whole manuscript is just narrative thus more suitable for a propaedeutic than a scientific journal. The cases and the presented fact do not add real new informations on the discussed dieseases. Some importand diagnoses like leucemia, metabolic diseases (like MPS I), Progressive pseudorheumatoid arthritis of childhood (PPAC) etc. aren´t even mentioned in the discussion. If the authors intend to summarize diagnoses leading to limping in childhood (apropos of 7 cases) than a more systemtic approach would be suitable for a Journal like PRo**.

We would like to thank the reviewer for his/her comment: there are various causes that can lead to limping; we have decided to discuss only those presented in this case series. However, we appreciate the suggestion to include some other important diseases; if the editorial office will agree we might be able to expand the manuscript. In the meantime, we have included a table (table 2) in which the all causes of limping are described, based on the etiology and divided by their frequency.

**Reviewer 2:**

**Dear authors, thanks for your manuscript. I think it's very useful for every pediatrician that has to consider other causes of chronic limping. I suggest to add the frequency of each disease both in text and table.
Thanks**

We would like to thank the reviewer. We appreciate his/her suggestion. We have therefore add the frequency of each disease both in text and table.

**Reviewer 3:**

**Thank you for overshadowing this common issue in childhood.
But some point needs to be considered:
1.      Arrangement of case scenario need to be modified to be in a systemic manner;
2.      The sentences in most of paper, need to modify in an essay way e.g
"Physical examination, there was a limitation of motion of the right hip and both wrists, with joints swelling. The physical examination was otherwise unremarkable''
      Can be Witten in a simple manner; the physical examination is unremarkable except for limitation of motion of right hip and both wrists**

We have tried to modify the manuscript in a systemic approach and the sentences of the paper as suggested.

**3. The discussion of cases; to less extant targeting the etiology with short notes on clinical picture and management in COPA syndrome case (1), unlike Neuroblastoma (Neuroblastoma) case focus only on clinical presentation without pointing to the etiology.**

We have standardized the discussion of the cases, adding the missing information

**4.    Case (6) Lyme arthritis need long history to present with arthritis after 2 years duration of exposure to tick bite, yes it was confirmed by serology, but detailed history to travel to endemic areas, other family exposure and so on.**

Regarding case (6), past medical history was negative. The boy had not traveled and did not remember a tick bite. We have corrected the typo that it was not 8 months but 2 months from the first episode.

**5.  You can reinforce the cases by mention a typical case of JIA, which as common diagnosis.**

We appreciate the suggestion and we added a typical case of JIA after all the other cases

**6. The figure must be followed by short description below it.**

A short description after each figure is below the references

**Reviewer 4:**

**It is an interesting manuscript. You should avoid abreviation JIA in the title**

As suggested, we changed the title, replacing JIA with juvenile idiopathic arthritis.

We highlighted changes in the manuscript.

Best regards,

Dr. Andrea Taddio
